# Supplementary material for: Absolute and Relative Strength, Power and Physiological Characteristics of Indian Junior National-Level Judokas
Source: Sports (Basel). 2020 Jan 28;8(2):14. doi: 10.3390/sports8020014 (PMC7077207; doi:10.3390/sports8020014)
Supplement: Supplementary file 1 [file sports-08-00014-s001.pdf]

sports

Supplementary Materials: Absolute and Relative Strength, Power and Physiological Characteristics of Indian Junior National-Level Judokas

Dale M Harris *, Kristina Kendall, G. Gregory Haff and Christopher Latella

Table S1. Participant demographics for each group. All values are presented as mean ± standard deviation. f, female; m, male.

Groups	Age (y) ± SD	Bodyweight (kg) ± SD	Height (cm) ± SD
Gold-medal winners (GM)<br>*(n = 8; m = 3, f = 5)*	Group = 16.4 ± 0.9<br>m = 16.3 ± 0.5<br>f = 16.4 ± 1.0	Group = 63.4 ± 10.1<br>m = 65.7 ± 1.9<br>f = 62.0 ± 12.5	Group = 161.0 ± 8.1<br>m = 170.3 ± 4.8<br>f = 155.4 ± 2.7
Gold, silver and bronze medal winners (MW)<br>*(n = 13; m = 6, f = 7)*	Group = 16.5 ± 1.4<br>m = 17.0 ± 0.8<br>f = 16.1 ± 1.6	Group = 63.2 ± 9.4<br>m = 67.5 ± 2.4<br>f = 59.6 ± 11.3	Group = 162.8 ± 9.9<br>m = 172.5 ± 5.1<br>f = 154.4 ± 3.2
Non-medallists (NM)<br>*(n = 12; m = 9, f = 3)*	Group = 16.6 ± 1.7<br>m = 16.7 ± 1.3<br>f = 16.3 ± 1.9	Group = 70.7 ± 9.9<br>m = 73.6 ± 5.3<br>f = 62.0 ± 12.7	Group = 168.2 ± 10.6<br>m = 172.8 ± 4.9<br>f = 154.3 ± 58.4
Silver and bronze medallists, and non-medallists (AO) *(n = 17; m = 12, f = 5)*	Group = 16.6 ± 1.8<br>m = 16.9 ± 1.3<br>f = 16.0 ± 2.5	Group = 68.4 ± 10.0<br>m = 72.5 ± 5.0<br>f = 58.6 ± 12.0	Group = 167.4 ± 11.1<br>m = 173.3 ± 4.8<br>f = 153.4 ± 9.0
Males (all)<br>*(n=15)*	16.8 ± 1.2	71.1 ± 5.3	170.7 ± 5.0
Females (all)<br>*(n=10)*	16.2 ± 1.9	60.3 ± 12.4	154.4 ± 6.7

Table S2. Mean and standard deviation (SD), percentage (%) difference and *p*-values for gold-medal winners (GM) compared to podium finishers and non-medallists (AO). * indicates significant difference ($p < 0.05$). CMJ, countermovement jump; 1-RM, one repetition maximum; BW, bodyweight; W, watts.

**Test**	**Mean ± SD**		**% diff**	**P-value**	**Hedge's g (95% CI)**
	**GM (n=8)**	**AO (n=17)**			
Handgrip strength (kg)	55.9 ± 2.9	56.4 ± 5.3	−0.7	0.838	0.09 (−0.93, 0.75)
Handgrip strength (kg/BW)	0.9 ± 0.1	0.8 ± 0.1	1.0	0.852	0.08 (−0.76, 0.92)
1-RM bench press (kg)	74.4 ± 16.8	71.2 ± 16.6	4.5	0.669	0.18 (−0.66, 1.02)
1-RM bench press (kg/BW)	1.14 ± 0.34	1.06 ± 0.25	6.8	0.550	0.28 (−0.57, 1.12)
1-RM back squat (kg)	116.1 ± 16.8	96.8 ± 23.2	20.0	0.046*	0.87 (−0.01, 1.74)
1-RM back squat (kg/BW)	1.75 ± 0.28	1.45 ± 0.36	21.0	0.047*	0.87 (−0.01, 1.74)
Aerobic power ($ml·kg·min^{-1}$)	37.4 ± 4.1	40.6 ± 6.8	−7.9	0.233	−0.51 (−1.36, 0.34)
Anaerobic power (W)	784.5 ± 49.7	625.8 ± 135.5	25.4	0.004*	1.32 (0.40, 2.24)
Anaerobic power (W/kg)	11.9 ± 1.7	9.3 ± 2.0	27.3	0.005*	1.27 (0.36, 2.18)
5-0-5 left (s)	1.5 ± 0.4	1.6 ± 0.3	−8.6	0.302	0.37 (−0.48, 1.22)
5-0-5 right (s)	1.4 ± 0.2	1.5 ± 0.3	−9.1	0.226	0.59 (−0.26, 1.45)
30m flying sprint (s)	3.9 ± 0.3	4.4 ± 0.8	−11.9	0.097	0.96 (0.08, 1.85)
CMJ power (W)	3049.6 ± 556.1	3016.1 ± 689.6	1.1	0.922	0.05 (−0.79, 0.89)

Table S3. Mean and standard deviation (SD), percentage (%) difference and *p*-values for medal-winners (MW) compared to non-medallists (NM). * indicates significant difference ($p < 0.05$). CMJ, countermovement jump; 1-RM, one repetition maximum; BW, bodyweight; W, watts.

**Test**	**Mean ± SD**		**% diff**	**P-value**	**Hedge's g (95% CI)**
	**MW (n=13)**	**NM (n=12)**			
Handgrip strength (kg)	57.6 ± 3.9	54.8 ± 5.0	5.2	0.126	0.61 (−0.19, 1.42)
Handgrip strength (kg/BW)	0.9 ± 0.1	0.8 ± 0.1	3.7	0.467	0.30 (−0.49, 1.09)
1-RM bench press (kg)	76.2 ± 16.5	67.9 ± 15.9	12.1	0.217	0.50 (−0.36, 1.35)
1-RM bench press (kg/BW)	1.1 ± 0.3	1.0 ± 0.2	10.9	0.316	0.42 (−0.43, 1.27)
1-RM back squat (kg)	110.7 ± 19.7	94.6 ± 24.0	17.0	0.079	0.68 (−0.18, 1.55)
1-RM back squat (kg/BW)	1.7 ± 0.4	1.4 ± 0.3	16.2	0.111	0.66 (−0.20, 1.52)
Aerobic power ($ml{\cdot}kg{\cdot}min^{-1}$)	38.3 ± 6.1	40.9 ± 6.3	−6.4	0.302	−0.41 (−1.25, 0.44)
Anaerobic power (W)	729.7 ± 142.3	619.1 ± 108.2	17.9	0.091	0.89 (0.02, 1.77)
Anaerobic power (W/kg)	10.9 ± 2.5	9.4 ± 1.7	16.3	0.091	0.75 (−0.12, 1.61)
5-0-5 left (s)	1.5 ± 0.3	1.6 ± 0.3	−3.6	0.645	0.17 (−0.66, 1.01)
5-0-5 right (s)	1.5 ± 0.3	1.6 ± 0.3	−1.8	0.816	0.09 (−0.78, 0.93)
30m flying sprint (s)	4.1 ± 0.6	4.4 ± 0.8	−6.7	0.324	−0.37 (−1.22, 0.48)
CMJ power (W)	3082.2 ± 613.6	2966.9 ± 686.2	3.9	0.634	0.17 (−0.61, 0.96)
